# Differentiated Thyroid Cancer: Indications and Extent of Central Neck Dissection—Our Experience

**DOI:** 10.1155/2013/625193

**Published:** 2013-09-26

**Authors:** Pietro Giorgio Calò, Fabio Medas, Giuseppe Pisano, Francesco Boi, Germana Baghino, Stefano Mariotti, Angelo Nicolosi

**Affiliations:** ^1^Department of Surgical Sciences, University of Cagliari, 09100 Cagliari, Italy; ^2^Department of Medical Sciences “Mario Aresu”, University of Cagliari, 09100 Cagliari, Italy

## Abstract

The aim of this retrospective study was to determine the rate of metastases in the central neck compartment and examine the morbidity and rate of recurrence in patients with differentiated thyroid cancer treated with or without a central neck dissection. Two hundred and fifteen patients undergoing total thyroidectomy with preoperative diagnosis of differentiated thyroid cancer, in the absence of suspicious nodes, were divided in two groups: those who underwent a thyroidectomy only (group A; *n* = 169) and those who also received a central neck dissection (group B; *n* = 46). Five cases (2.32%) of nodal recurrence were observed: 3 in group A and 2 in group B. Tumor histology was associated with a risk of recurrence: Hürthle cell-variant and tall cell-variant carcinomas were associated with a high risk of recurrence. Multifocality and extrathyroidal invasion also presented a higher risk, while smaller tumors were at lower risk. The results of this study suggest that prophylactic central neck dissection should be reserved for high-risk patients only. A wider use of immunocytochemical and genetic markers to improve preoperative diagnosis and the development of methods for the intraoperative identification of metastatic lymph nodes will be useful in the future for the improved selection of patients for central neck dissections.

## 1. Introduction

Differentiated thyroid carcinoma (particularly papillary) is the most common thyroid malignancy, comprising approximately 90% of new cases of thyroid cancer in iodine-sufficient areas of the world [1–3]. The prognosis of treated differentiated thyroid cancer is generally excellent, with 10-year overall survival rates exceeding 90% [1, 2, 4]. Total thyroidectomy is generally accepted as the procedure of choice for all papillary thyroid carcinomas exceeding 10 mm in diameter [1, 2].

Lymph node metastases are common in papillary thyroid carcinoma, occurring in 20–50% of patients, as identified using standard pathological techniques [1, 2, 5–10]. Micrometastases are even more common and may be found in 90% of patients [5–7, 10]. The central compartment of the neck, also known as level VI, is the most frequently involved [1, 2, 5, 8, 11–13]. 

Regional lymph node involvement is associated with increased tumor recurrence [2, 5, 7, 14], and recurrence rates are higher in node positive patients over the age of 45 years [5].

When neither imaging nor palpation of the lymph nodes arouses the suspicion of metastatic disease, the value of prophylactic central neck dissection becomes a matter of debate [2, 15]. It has traditionally been accepted that regional lymph node metastases in papillary thyroid carcinoma may increase regional recurrence rates but do not ultimately affect survival rates [7, 9, 10, 14], although some recent studies have suggested a possible role of lymph node metastasis from papillary thyroid cancer in reducing patient's survival [15, 16].

Some authors recommend routine central neck dissection in order to prevent a future recurrence, citing the high risk of positive lymph nodes, better outcomes, and a lower morbidity rate associated with this initial operation, whereas others maintain that this procedure increases the risk of injury to the parathyroid glands and recurrent laryngeal nerves, without any demonstrable benefits in terms of long-term survival [2, 6, 13, 15]. Central neck dissection allows for accurate pathologic staging of the lymph nodes and treatment of the micrometastases that may be responsible for the recurrence or persistence of the disease [15]. In addition, recurrence in this compartment is difficult to treat surgically and may become complicated [11, 17], while central lymph node dissection during the initial thyroid surgery can be performed without extending the wound [17].

Neck ultrasonography (US), the accuracy of which is increased by use of fine-needle aspiration cytology (FNAC) and FNAC thyroglobulin (Tg) measurements, reliably evaluates the lateral compartment of the neck before thyroid surgery [15, 18]. This procedure can detect nonpalpable lymph node metastases, thereby helping to plan therapeutic lateral dissections combined with total thyroidectomy [15, 18]. In contrast, no completely reliable tool currently exists for the preoperative detection of lymph node metastases in the central compartment of the neck [15]. The accuracy of neck ultrasonography is limited by the fact that paratracheal lymph nodes are minute and located beneath the thyroid gland. The air-filled trachea provides an additional element of disturbance [15].

The aim of this retrospective study was to determine the rate of metastases in the central neck and examine the morbidity and rate of recurrence in patients with differentiated thyroid cancer, treated with or without a central neck dissection.

## 2. Materials and Methods

The clinical records of patients undergoing total thyroidectomy with or without prophylactic lymphadenectomy, between 2002 and 2008, presenting preoperative cytological evidence of differentiated thyroid cancer but no signs of enlarged lymph nodes during preoperative ultrasonography and intraoperative inspection and palpation were analyzed. In our unicenter retrospective study, 215 consecutive differentiated thyroid cancer patients were identified by computerized search. These patients had undergone total thyroidectomy with curative intent in our Department of Surgical Sciences at the University of Cagliari. Patients were divided into two groups: those who had received a thyroidectomy without central neck dissection (group A; *n* = 169) and those who had receveid a thyroidectomy with central neck dissection (group B; *n* = 45). These patients were considered for central neck dissection on the basis of macroscopic appearance of the tumor, tumor size, and suspicion of extracapsular invasion. In all cases, surgery was performed by three experienced endocrine surgeons. For each patient, a preoperative diagnosis of differentiated thyroid cancer had been obtained by ultrasound (US)-guided FNAC. The preoperative workup consisted of free thyroid hormone (FT3, FT4), thyrotropin (TSH), Tg and anti-Tg antibody (TgAb) measurements, and high resolution US of the neck by a skilled sonographer. A pre- and postoperative fibrolaryngoscopy was routinely performed in all patients. Postoperative diagnosis of lymph node recurrence was performed by US-guided FNAC and Tg measurement in FNAC fluid washout (FNAC-Tg) in the case of enlarged lymph nodes ≥ 1 cm, as previously described [19]. Patient demographics and postoperative complications were recorded, including neck hematomas requiring reoperation, transient or permanent hypoparathyroidism, transient or permanent vocal cord palsy, and distant and locoregional recurrence detected by postoperative surveillance. Hypoparathyroidism (defined as a PTH level < 10 pg/mL; normal values range between 10 and 65 pg/mL) was considered permanent when it lasted for more than 6 months. Qualitative data were expressed as percentages, while quantitative data were expressed as the mean value ± standard deviation.

### 2.1. Biochemical Assays

FT3, FT4, and TSH were determined by automatic ultrasensitive chemiluminescent assays (Ortho-Clinical Diagnostic SpA, Milan, Italy). Tg and TgAb were detected by chemiluminescent assays (Immulite 2000, Diagnostic Products Corporation, Los Angeles, CA, USA; distributor: Medical Systems Corporation, Genoa, Italy).

### 2.2. Surgery

All operations were performed by the same three surgeons; thus, the total thyroidectomy and lymph node dissection techniques used were consistent across patients. Recurrent laryngeal nerves were routinely identified and exposed up until their insertion in the larynx, and parathyroid glands were identified and preserved. In cases of suspected devascularized or incidentally removed parathyroid glands, a muscular autoimplantation followed. Serum calcium and intact parathyroid hormone levels were assayed on the first postoperative day and subsequently on the basis of a clinical evaluation.

### 2.3. Radioiodine Ablation

After surgery, 197 patients (91.62%) underwent adjuvant radioiodine ablation (1,850–3,700 MBq-^131^I). Apart from lymph node involvement, indications for postoperative ^131^I treatment were a tumor >1 cm, extracapsular thyroid invasion or locoregional extension, unfavorable histological subtype (follicular, diffuse sclerosing, or tall cell-variant papillary cancer), multifocal disease, or BRAF positive tumor specimens. 18 patients (8.37%) with tumor <1 cm and without risk factors (lymph node involvement, extracapsular invasion, unfavorable histological subtype, multifocal disease, or BRAF positive) were not submitted to adjuvant radioiodine ablation. To obtain adequate levels of endogenous TSH (>30 mU/mL) that are associated with an increased radioiodine uptake, patients stopped L-T4 replacement 3-4 weeks before radioiodine treatment; when L-T4 withdrawal was not indicated, TSH stimulation was achieved using Recombinant Human Thyrotropin (rhTSH, Thyrogen), according to standard protocols. Posttherapy whole-body scanning was performed 4–7 days after radioiodine treatment.

### 2.4. Followup

Patient follow-up examinations consisted of neck ultrasounds and the monitoring of serum Tg and TgAb levels every 6 months during suppressive L-thyroxine treatment. A serum Tg level ≤ 0.2 ng/mL was taken as undetectable. Surveillance for possible recurrence in patients considered that disease free was achieved by Tg detection or, in those positive for TgAb, by whole-body ^131^I scanning after rhTSH stimulation and neck ultrasound. Diagnosis of disease recurrence in the cervical lymph nodes was based on US-guided FNC, Tg washing of FNC aspirates, and serum Tg level monitoring. The mean length of followup was 93 months (range 55–130 months).

### 2.5. Statistical Analysis

Data were analyzed using descriptive statistics: for categorical variables, the Pearson's chi-squared (exact) test was used; for quantitative variables, independent Student's *t*-tests were used. Data were reported as the mean value ± standard error of the mean (SEM). All calculations were performed using the software package GraphPad Prism, Version 5.0 for Windows (GraphPad Software, San Diego, CA, USA). Comparisons were considered statistically significant for *P* < 0.05.

## 3. Results

Between September 2002 and December 2008, 215 differentiated thyroid cancer patients, 173 women and 42 men (F/M ratio = 4.97/1), with a mean age of 51.2 ± 14.34 years (range 18–81), were submitted to total thyroidectomy. Group A consists of 169 patients, 135 female and 34 male (F/M ratio = 3.97/1), with a mean age of 52.3 ± 14 years (range 18–81), who were submitted to total thyroidectomy without lymphadenectomy. Group B consists of 46 patients, 38 female and 8 male (F/M ratio = 4.75/1), with a mean age of 47.15 ± 15.01 (range 20–77), who were submitted to total thyroidectomy with prophylactic central neck lymphadenectomy (see Table 1). A neck hematoma, requiring surgical reexploration, was observed in 2 patients (0.93%): one in group A (0.59%) and one in group B (2.17%) (*P* = 0.9). Transient or definitive hypoparathyroidism was observed in 33 (19.52%) and 8 patients (4.73%), respectively, in group A; while 15 (32.60%) and 5 patients (10.86%) were diagnosed in group B, respectively (*P* = 0.091 and 0.23). Temporary recurrent laryngeal nerve paralysis was observed in 2 patients in group A (1.18%) and in 2 patients in group B (4.34%) (*P* = 0.42). No cases of permanent or bilateral recurrent laryngeal paralysis were observed (see Table 2).

### 3.1. Pathological Data

Mean tumor size was 15.33 mm (15.1 mm in group A and 16.17 mm in group B). A microcarcinoma (<1 cm) was diagnosed in 48 patients (22.32%): 41 in group A (24.26%) and 7 in group B (15.21%). The histotype was classic papillary in 135 patients (62.79%, 102 in group A and 33 in group B), follicular variant in 52 (24.65%, 42 in group A and 11 in group B), follicular in 18 (8.37%, 16 in group A and 2 in group B), Hürthle cell variant in 5 (2.32%, all in group A), and tall cell variant in 4 (1.86%, all in group A). 62 patients (28.83%) had multifocal tumors: 44 in group A (26.03%) and 18 in group B (39.13%). 52 patients (24.18%) had a locoregional infiltration (T3): 32 in group A (18.93%) and 20 in group B (43.47%) (see Table 1).

The 2 groups were well matched for sex, histology, tumor size, multifocality, and incidence of microcarcinomas, while the mean age was slightly higher in group A, and locoregional infiltration was most represented in group B. In group B, metastases were found in 12 patients (26.08%) and nodal micrometastases in 2 cases (4.34%). The mean number of removed lymph nodes was 7.6. In patients with metastases, the mean number of metastatic nodes was 2.9.

### 3.2. Recurrence

No patient developed distant recurrence during followup. After total thyroidectomy and radioiodine ablation, ipsilateral nodal (III-IV) recurrence was observed in 5 cases (2.32%): 3 in group A (1.77%) and 2 in group B (4.34%) (*P* = 0.63). No central (VI) node recurrences were observed. The demographic characteristics of patients with recurrence were the following: 1 male (47 years) and 4 females (median age 57.5 years; range 34–69). The mean elapsed time between intervention and lymph node recurrence was 12 months in group A (range 6–18) and 7.5 months in group B (6 and 9). One patient had classic-variant, 1 had follicular-variant, 1 Hürthle cell-variant, and 2 patients had tall-cell variant papillary carcinomas. In particular, this last variant was strongly associated with recurrence (*P* < 0.001; Table 3). Locoregional infiltration and tumor size were also associated to risk of recurrence (*P* = 0.015 and 0.046, respectively, Table 3). In all cases, a lateral and central lymph node dissection was performed, followed by another session of radioiodine ablation. The mean number of removed nodes was 4; the mean number of metastatic nodes was 2.25. No complications were observed after reoperation.

## 4. Discussion 

Subclinical nodal disease is found histopathologically in the majority of patients with differentiated thyroid cancer; however, the management and impact on the prognosis of this form of lymph node metastasis are unclear [6]. The most commonly involved lymph nodes in thyroid carcinoma are the central nodes. Nodal involvement in differentiated thyroid cancer has been shown in a number of retrospective studies to be associated with an increased risk of locoregional recurrence but not with overall survival [2, 6, 15, 20, 21]. Other reports indicate that nodal dissection in differentiated thyroid cancer can advantageously decrease locoregional recurrence and improve survival [2, 6, 15, 21]. 

Arguments in favor of prophylactic central node dissection are as follows: the high number of lymph node metastases; the insufficient diagnostic accuracy of ultrasonography and intraoperative exploration in 1/3 of differentiated thyroid cancer patients; and the failure of ^131^I ablation in about 30% of cases, especially when an enlarged lymph node greater than 1 cm is present [6, 22].

Metastases in level VI nodes in papillary thyroid cancer are common, with macroscopically positive gross nodal disease being present in 10–30% of patients [6]; while the incidence of clinically nonpalpable disease is reported in 40–70% of patients [6, 14]. Moreover, studies show that 70% to 90% of patients with papillary thyroid cancer present no evidence of lymph node metastasis, whilst being positive for micrometastases in adjacent lymph nodes [6, 9, 14, 17]. Shen [13] reported a lymph node recurrence rate of 10–15% in patients who did not undergo routine lymph node dissection [6].

High resolution cervical US is reportedly the most sensitive method for differentiating between metastatic and benign lymph nodes in patients with differentiated thyroid cancer and for detecting locoregional metastases as small as 2-3 mm in dimension. Thus, in a significant proportion of patients with small macroscopic metastases, preoperative US can be used to detect metastatic lymph nodes that may be missed by palpation alone. Unfortunately, although US has a high sensitivity and specificity in detecting lateral lymph node metastasis, it cannot be used for accurately detecting lymph node metastases in the central neck compartment [4, 19, 23]. Several studies showed that the sensitivity of ultrasonography and computed tomography was not accurate enough for the staging of central lymph nodes, ranging between 50 and 70% [2]. Moreover, preoperative ultrasonography was found to have high specificity and a positive predictive value but low sensitivity and a negative predictive value (of only about 61%) for the detection of lymph node metastases in the central neck compartment [22, 24]. 

Central node dissection increases the number of patients with undetectable Tg levels [6]. For example, one study found that the number of patients with basal Tg serum levels < 0.2 ng/mL during levothyroxine suppression therapy was significantly higher among those who had undergone prophylactic central neck dissection [2]. Indeed, the addition of central lymph node dissection to total thyroidectomy is repeatedly reported to decrease serum levels of Tg and increase the rates of undetectable Tg [1, 10, 11].

Another argument in favor of prophylactic central neck dissection is the lower associated rates of morbidity compared to those associated with reoperation [6, 25]. In fact, reoperative central lymph node dissection has the potential to be more challenging and put the recurrent laryngeal nerve and parathyroid glands at increased risk [12, 24] due to increased scar tissue. Indeed, scarring, edema, and friability of the tissues together with distortion of the landmarks make reoperative surgery hazardous [26], and reoperative surgery has also been associated with a higher risk of postoperative hematoma [27].

A prophylactic central lymphadenectomy permits a better staging of central neck compartment lymph nodes, but further benefits remain to be demonstrated [6]. Authors report a 30% increase in the number of patients with T1 differentiated thyroid cancers (preoperatively considered to be N0), for whom ^131^I ablation was indicated following routine central and lateral nodal dissection demonstrating unexpected nodal metastases. The rational for this approach, in patients with tumors < 1 cm, is that positive lymph nodes are an indication for radioiodine ablation [6]. Lymphadenectomy seems to play a role in staging and may therefore be indicated when radioactive treatment would not normally be administered. When radioiodine treatment is advisable, routine lymph node dissection does not modify the treatment protocol and is, therefore, not indicated [6]. Recent studies have suggested that approximately one-third of patients who have prophylactic central neck dissection may be upstaged [2, 25], and as a consequence radioiodine therapy is used significantly more frequently in these patients [2]. Thus, patients undergoing central neck dissection also have a higher chance of receiving treatment for subclinical micrometastatic disease [2]. Indeed, prophylactic central neck dissection was found to result in an increased use of radioactive ^131^I and, ultimately, in more favorable outcomes [2].

Many endocrine surgeons doubt that routine lymph nodal dissection offers any real benefits to patients, arguing that the procedure is associated with higher morbidity, especially injury to the parathyroid gland (most frequently the lower glands) [6, 12, 15], with rates of transient hypoparathyroidism of 14–60%, permanent hypoparathyroidism of 3–11%, transient vocal cord paralysis of 3–7%, and permanent recurrent laryngeal nerve injury of 0–4% [11, 14, 28–30]. Total thyroidectomy is associated with a low morbidity rate, and due to radioiodine treatment and TSH suppression therapy, the incidence of locoregional lymph node recurrence is low. The need for reoperation in the central compartment is uncommon, but it is generally accepted that reoperations in the cervical compartments are associated with greater risks of hypoparathyroidism and recurrent nerve palsy [1, 31, 32]. However, Kim et al. [33] reported a 0% incidence of new recurrent laryngeal nerve palsy and a 0.4% incidence of permanent hypoparathyroidism in a series of 20 patients who underwent reoperation of the central lymph node dissection for recurrent or persistent thyroid cancer, data which has been confirmed by other authors [1]. Reoperation (lymph node dissection) is not usually associated with higher morbidity, especially in cases of unilateral dissection, although hypoparathyroidism and recurrent laryngeal nerve injury have been observed in up to 14% and 9% of patients, respectively [6].

The low rate (2.32%) of lymph node recurrence following total thyroidectomy observed in the present study is in line with data reported by others [6]. We did not observe any complications after reoperations, likely because of the small number of patients requiring a second surgical intervention.

The complication rates observed here following prophylactic central neck dissection (group B) are, in our experience, high; indeed, the incidence of complications is approximately double compared to those undergoing thyroidectomy only (Group A), even if the differences between the two groups do not reach statistical significance, probably because of the limited number of cases. In particular, the rate of temporary hypoparathyroidism stands at 32.6% versus 19.52% (*P* = 0.09).

The issue of unilateral versus bilateral central neck dissection continues to be another area of controversy [2]. Ipsilateral central neck dissection may be sufficient for tumors measuring 1 cm or less [2] and therefore avoids the risk of complications arising from bilateral central neck dissection [6, 15]. Nevertheless, a bilateral central neck dissection was performed in all the 46 cases of the present study, in view of the risk of skip and contralateral metastases [34].

As a matter of fact, the recent American Thyroid Association Guidelines, as well as a meta-analysis conducted by Chisholm et al. [5], whilst taking into consideration all the pros and cons, recommend routine central lymph node dissection for all differentiated thyroid cancers, especially in high-risk patients [6, 15].

However, prophylactic central neck dissection is not recommended for low-volume thyroid surgeons; in fact, the risk of nerve injury and hypocalcaemia appears to be significantly greater for low-volume centers [2].

Of consequence, the current indications for performing a prophylactic central neck dissection are still a matter of debate. Our low recurrence rate (2.32%) combined with the nonnegligible incidence of complications (particularly transient and definitive hypoparathyroidism, 32.60% and 10.86%, resp.) leads us to sustain that prophylactic central lymph node dissection should not be carried out on a routine basis in the treatment of differentiated thyroid cancer patients. Instead, it may be more useful to develop criteria for the identification of high-risk patients for whom central neck dissection could be of benefit. The incidence of recurrence depends on numerous factors, such as tumor size, patient age, sex (males are more disposed to recurrence), and extracapsular spread [2, 5, 15]. In our study, tumor histology was strongly associated with the risk of recurrence: Hürthle cell-variant (20%) and, in particular, tall cell-variant (50%) carcinomas were associated with a high risk of relapse (*P* < 0.001). Multifocality (6.45%, *P* = 0.039) and extrathyroidal invasion (7.69%, *P* = 0.015) also presented a higher risk of recurrence, in line with other reports in the literature [2, 5, 15]. We found no differences in relation to age or gender, while smaller tumors were at lower risk of recurrence, in agreement with other reports in the literature [2, 5, 15]. Moreover, 22.32% patients presented microcarcinomas (tumors ≤ 1 cm in diameter) with low risk of recurrence, a rate that is again in agreement with the literature [35].

The problem that remains is how to define the assessment criteria of high-risk patients, considering the fact that only the size of the tumor can be assessed preoperatively, while the type and the histological characteristics (i.e., locoregional infiltration and multifocality) can usually only be identified after surgery. We believe that a prophylactic central neck dissection should not be routinely recommended for smaller tumors (≤1 cm), while it may be advisable for larger tumors (>2 cm), especially if cytological suspicion of a high-risk subtype arises or if there are intraoperative signs of extracapsular spread. A wider use of immunocytochemical and genetic markers could prove useful in better defining the high-risk population. For example, patients with RET/PTC oncogene expression have a higher rate of lymph node metastases [36], and this could constitute a useful factor to consider in the future. The development of techniques for the intraoperative identification of metastatic lymph nodes could also help the surgeon in this difficult choice.

## 5. Conclusions

The role of prophylactic central lymph node dissection in the management of differentiated thyroid cancer is controversial. No conclusive evidence exists to indicate that a prophylactic central neck dissection has a beneficial effect on recurrence or mortality rates. The recurrence rate found in this study of 2.32% confirms the rarity of lymph node recurrence and leaves many doubts regarding the usefulness of prophylactic central neck dissection.

Prophylactic central neck dissection is associated with increased morbidity, even when performed by experienced surgeons; in particular, it is associated with a higher rate of transient complications, mostly hypoparathyroidism, while the rate of permanent complications is very low and not significantly different from that for total thyroidectomy alone. Our study confirms an increase in the complication rate when a central neck dissection is also performed, although the data do not achieve statistical significance, most probably due to the small sample size.

To summarize, we believe that prophylactic central neck dissection should be reserved for high-risk patients only. Unfortunately, no clinical or pathological factors are able to predict with any certainty the presence of nodal metastasis. However, in our experience, tumor size is related to an increased risk of recurrence, as are some histological types (Hürthle cell and, particularly, tall cell variant), multifocality, and locoregional infiltration.

A wider use of immunocytochemical and genetic markers could be useful in improving the preoperative diagnosis, and the development of methods to aid the intraoperative identification of metastatic lymph nodes will certainly be useful in the near future for the improved selection of patients for prophylactic central neck dissection.

## Figures and Tables

**Table 1:** Demographic and pathological data of 215 differentiated thyroid cancer patients.

	Total	Group A	Group B	*P*
Patients	215	169	46	
Male	42 (19.53%)	34 (20.11%)	8 (17.39%)	0.8384
Female	173 (80.46%)	135 (79.88%)	38 (82.60%)	
Mean age (years)	51.2 ± 14.34	52.3 ± 14	47.15 ± 15.01	0.0303
Histology				
Papillary classic	135 (62.79%)	102 (60.35%)	33 (71.73%)	0.36
Follicular variant	53 (24.65%)	42 (24.85%)	11 (23.91%)	
Hürthle cell variant	5 (2.32%)	5 (2.95%)	0	
Tall cell variant	4 (1.86%)	4 (2.36%)	0	
Follicular	18 (8.37%)	16 (9.46%)	2 (4.34%)	
Tumor				
Mean size (mm)	15.33	15.1	16.17	0.5215
Unique	153 (71.16%)	125 (73.96%)	28 (60.86%)	0.12
Multifocal	62 (28.83%)	44 (26.03%)	18 (39.13%)	
Microcarcinoma	48 (22.32%)	41 (24.26%)	7 (15.21%)	0.2686
Locoregional infiltration	52 (24.18%)	32 (18.93%)	20 (43.47%)	0.0011

**Table 2:** Total thyroidectomy: complications.

	Total	Group A	Group B	*P*
Temporary hypoparathyroidism	48 (22.32%)	33 (19.52%)	15 (32.60%)	0.091
Permanent hypoparathyroidism	13 (6.04%)	8 (4.73%)	5 (10.86%)	0.23
Temporary unilateral vocal cord palsy	4 (1.86%)	2 (1.18%)	2 (4.34%)	0.42
Permanent unilateral vocal cord palsy	0	0	0	—
Bilateral vocal cord palsy	0	0	0	—
Neck hematoma	2 (0.93%)	1 (0.059%)	1 (2.17%)	0.9

**Table 3:** Risk factors of recurrence: gender and histology.

	Recurrence	*P*
Gender		
Male	1/42 (2.38%)	0.58
Female	4/173 (2.31%)	
Mean age	53 ± 15.11 (versus 51.16 ± 14.36 non recurrent)	0.77
Histology		
Papillary classic	1/135 (0.74%)	*P* < 0.001
Follicular variant	1/53 (1.88%)	
Hürthle cell variant	1/5 (20%)	
Tall cell variant	2/4 (50%)	
Tumor		
Mean size	24.6 ± 4.66 (versus 15.11 ± 10.54 non recurrent)	0.046
Unique	1/153 (0.65%)	0.039
Multifocal	4/62 (6.45%)	
Locoregional infiltration		
Present	4/52 (7.69%)	0.015
Absent	1/163 (0.61%)	
